# Cutaneous Granulomas in Dolphins Caused by Novel Uncultivated *Paracoccidioides brasiliensis*

**DOI:** 10.3201/eid2212.160860

**Published:** 2016-12

**Authors:** Raquel Vilela, Gregory D. Bossart, Judy A. St. Leger, Leslie M. Dalton, John S. Reif, Adam M. Schaefer, Peter J. McCarthy, Patricia A. Fair, Leonel Mendoza

**Affiliations:** Federal University of Minas Gerais, Belo Horizonte, Brazil (R. Vilela);; Michigan State University, East Lansing, Michigan, USA (R. Vilela, L. Mendoza);; Georgia Aquarium, Atlanta, Georgia, USA (G.D. Bossart);; University of Miami Miller School of Medicine, Miami, Florida, USA (G.D. Bossart);; SeaWorld, San Diego, California, USA (J.A. St. Leger);; SeaWorld, San Antonio, Texas, USA (L.M. Dalton);; Colorado State University College of Veterinary Medicine and Biomedical Sciences, Fort Collins, Colorado, USA (J.S. Reif);; Florida Atlantic University, Fort Pierce, Florida, USA (A.M. Schaefer, P.J. McCarthy);; National Oceanic and Atmospheric Administration, Charleston, South Carolina, USA (P.A. Fair)

**Keywords:** lacaziosis, lobomycosis, cutaneous granulomas, Lacazia loboi, Paracoccidioides brasiliensis, P. lutzii, fungi, paracoccidioidomycosis, paracoccidioidomycosis ceti, bottlenose dolphins, Tursiops truncatus, dolphins, phylogenetic analysis

## Abstract

Cutaneous granulomas in dolphins were believed to be caused by *Lacazia loboi*, which also causes a similar disease in humans. This hypothesis was recently challenged by reports that fungal DNA sequences from dolphins grouped this pathogen with *Paracoccidioides brasiliensis*. We conducted phylogenetic analysis of fungi from 6 bottlenose dolphins (*Tursiops truncatus*) with cutaneous granulomas and chains of yeast cells in infected tissues. *Kex* gene sequences of *P. brasiliensis* from dolphins showed 100% homology with sequences from cultivated *P. brasiliensis*, 73% with those of *L. loboi*, and 93% with those of *P. lutzii.* Parsimony analysis placed DNA sequences from dolphins within a cluster with human *P. brasiliensis* strains. This cluster was the sister taxon to *P. lutzii* and *L. loboi*. Our molecular data support previous findings and suggest that a novel uncultivated strain of *P. brasiliensis* restricted to cutaneous lesions in dolphins is probably the cause of lacaziosis/lobomycosis, herein referred to as paracoccidioidomycosis ceti.

## Abstract 2

Our findings could stimulate study of public health implications of diseases caused by this fungus.

The clinical and phenotypic features of the uncultivated agent of lacaziosis/lobomycosis in dolphins suggested that this pathogen was the same organism as *Lacazia loboi*, which causes skin keloidal-like lesions in humans (*1*–*6*). Although several studies indicated that *L. loboi* from human resists culture (*4*–*6*), only 1 well-documented study shows the uncultivated nature of the pathogen causing cutaneous granulomas in dolphins (*7*). Thus, the true ecology, epidemiology, and taxonomy of these 2 uncultivated pathogens of humans and dolphins have been controversial (*4*,*7*).

Because of their phenotypic resemblance and serologic cross-reactivity with *Paracoccidioides brasiliensis*, at one time these pathogens were believed to be *P. loboi* (*4*,*8*). This taxonomic controversy was partially resolved in 1999 when Taborda et al. (*9*) proposed the binomial *L. loboi* and concluded that previous terms used to name the etiologic agent of skin keloidal-like lesions in humans and dolphins were invalid. Molecular analysis of internal transcriber spacer (ITS) and chitin synthase 4 (*CHS4*) genes validated their original proposal (*10*). Further phylogenetic analysis of several genomic DNA sequences showed that *L. loboi* was closely related to *Paracoccidioides* spp. (*11*). However, other molecular data showed that *L. loboi* from humans was located in its own genus because of strong bootstrap support (*12*).

The notion that human *L. loboi* was the same organism as those in the skin of dolphins with lacaziosis/lobomycosis was first challenged by Rotstein et al. (*13*), who used molecular analysis. These investigators found that the 28S rDNA amplicon of *L. loboi* in extracted genomic DNA from an infected bottlenose dolphin (*Tursiops truncatus*) in North Carolina, USA, coastal areas had 97% identity with *P. brasiliensis* DNA sequences available in GenBank. However, their DNA sequences are not available. More recently, 3 groups in Japan (*14*,*15*) and Spain (*16*), who also used molecular methods, reported similar observations for several dolphin species including, *T. truncatus* and *Lagenorhynchus obliquidens*, which had skin granulomas and yeast-like cells in infected tissues. These studies showed that glycoprotein 43 (*gp43*)–like and ITS partial DNA sequences isolated from infected dolphins placed the etiologic agent of skin granulomas among human *P. brasiliensis* strains.

We amplified by using PCR the partial coding DNA sequences of the *Kex* gene in genomic DNA isolated from 6 bottlenose dolphins with cutaneous granulomas. These dolphins were captured in the Indian River Lagoon, Florida, USA, a 156-mile estuary along the eastern coast of the United States. Phylogenetic analysis showed that *Kex* PCR amplicons, which contained partial DNA sequences of the Kex protein, clustered among cultivated *P. brasiliensis* strains from humans with systemic paracoccidioidomycosis. Our data suggest that a novel uncultivated *P. brasiliensis* type, different from *L. loboi* from humans, is the probable etiologic agent of cutaneous granulomas in dolphins.

## Materials and Methods

### Biopsy Specimens from Bottlenose Dolphins

Four formalin-fixed tissues were received from the Harbor Branch Oceanographic Institute (Fort Pierce, FL, USA). Samples were collected in June 2003 from bottlenose dolphins captured in the Indian River Lagoon with cutaneous granulomas displaying chains of yeast cells in the infected tissues (FB 921, FB938, FB946, and FB952). Two additional skin biopsy specimens were obtained from SeaWorld of Texas (San Antonio, TX, USA); 1 specimen (SW070458) was collected during rescue and rehabilitation efforts, and a second 1 specimen (B92-932) was obtained from an animal that came from the Indian River Lagoon and was then kept at SeaWorld of Texas (Table).

**Table:** Uncultivated *Paracoccidioides brasiliensis* strains isolated from 6 bottlenose infected dolphins (*Tursiops truncatus*), Indian River Lagoon, Florida, USA

Strain	Dolphin age, y/sex	Year of collection
FB-921	Unknown/F	2003
FB-938	15/M	2003
FB-946	17/M	2003
FB-952	18/M	2003
B92–932	14/F	1992
SW070458	19/F	2007

### Isolation of DNA from Paraffin-Embedded Tissues

Using a sterile microtome, we obtained 10-mm–thick sections from paraffin-embedded tissues. Parts of sections were examined by using histopathologic analysis after staining with Gomori methenamine silver to verify the presence and quantity of yeast-like cells in selected specimens.

Isolation of DNA was performed by using the BioChain FFPET protocol (BioChain Institute, Inc., Newark, CA, USA). In brief, at least three 10-mm–thick sections were placed in a 1.5-mL microcentrifuge tube, and 500 μL of Dewaxil reagent was added. The sample was incubated at 90°C for 1 h, followed by addition of 180 μL of lysis buffer and a brief centrifugation. Two phases were formed; 20 μL of proteinase K was added to the lower phase, and the mixture was incubated at 56°C for 1.5 h. After incubation, the sample was centrifuged for 1 min, and the lower phase was transferred into a new tube. RNase A (2.0 μL, 100 mg/mL) was added, followed by addition of 100 μL of binding buffer and 100 μL of 100% ethanol. The entire mixture was then transferred into a separation column (BioChain Institute, Inc.) and centrifuged at 6,000 × *g* for 1 min. The column was washed twice with the provided buffers. DNA was extracted by adding 50 μL of elution buffer and centrifuging for 1 min at maximum speed. Samples were used immediately or stored at −80°C.

### Amplification and Sequencing of Partial *Kex* Gene Sequences

Because genomic DNA extraction from formalin-fixed tissues usually degrades genome DNA into small pieces, we designed primers targeting fragments <300 bp. To properly verify previous findings, we selected a conserved region of the *Kex* partial DNA sequence to target a DNA epitope other than *gp43* and ITS sequences used by other investigators (*14*–*16*). We used the protocol of Vilela et al. (*12*) to search for homologous DNA sequences of Kex protein in GenBank, aligned sequences by using ClustalW, version 1.81 (*17*), and inspected them visually.

Conserved regions were selected to construct the set of primers *Kex*-1F 5′-TGCTTYGGTTTGGGGTTG-3′ and *Kex*-2R 5′-CACTGGARCCGTCAGCTA-3′. The set of primers were designed to amplify a 151-bp region of the *Kex* DNA sequence according to the PCR protocol of Vilela et al. (*12*). Amplicons were ligated into the pCR 2.1-TOPO vector (Invitrogen, Carlsbad, CA. USA), purified, and sequenced by using BigDye Terminator Chemistry in an ABI Prim 310 Genetic Analyzer (Applied Biosystems, Foster City, CA. USA).

To further corroborate our results, we used *gp43* DNA sequences reported by Minakawa et al. (GenBank accession no. AB811031) (*14*) and Ueda et al. (GenBank accession no. LC067206) (*15*) and ITS DNA sequences reported by Esperón et al. (GenBank accession no. HQ413323) (*16*) for phylogenetic analysis of several homologous DNA sequences of *P. brasiliensis*, *P. lutzii*, and *L. loboi* in GenBank. We also analyzed 2 unpublished *CHS4* gene sequences (GenBank accession nos. KX267767 [A3] and KX267768 [90A]; A. Schaefer, P, McCarthy, unpub. data) isolated in 2008 from 2 dolphins with lacaziosis/lobomycosis in the Indian River Lagoon.

### Phylogenetic Analyses

Homologous DNA sequences of partial *CHS4, gp43*, *Kex*, and ITS sequences of *P. brasiliensis*, *P. lutzii*, *L. loboi*, *Ajellomyces capsulatus*, and *A. dermatitidis* were aligned by using default settings in ClustalW, version 1.81 (*17*) inspected visually, and exported for analysis by using maximum-parsimony and neighbor-joining in MEGA6 (http://www.megasoftware.net) (*18*). Aligned sequences were exported for parsimony analysis by using a heuristic search with tree bisection reconnection branch swapping (MEGA6) and distant analysis by neighbor-joining (MEGA6).

We coded large insertions as 1 event by excluding all but 1 nt/insertion. Generated gaps were treated as missing data. Neighbor-joining analyses used either uncorrected distances or maximum-likelihood estimates of distances with a general time reversible model (6ST), empirical base frequencies, and either no rate variation among sites or a gamma distribution (shape parameter 0.5) of variation among sites with 4 rate categories. Support for branches was estimated as the percentage of neighbor-joining trees containing the branch on the basis of neighbor-joining analysis of maximum likelihood distances of 1,000 bootstrapped datasets.

## Results

### PCR Amplification and Analysis by Using Basic Local Alignment Search Tool

**Figure 1:**
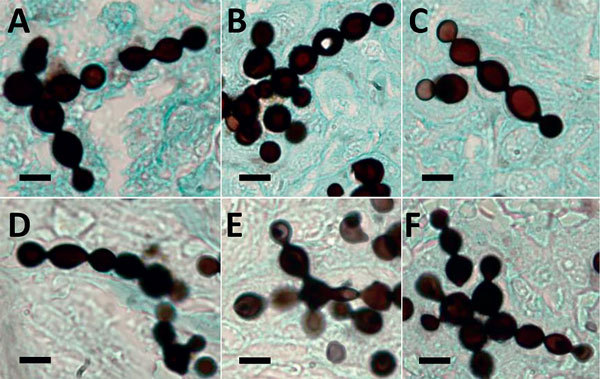
Infected tissues from 6 bottlenose dolphins (*Tursiops truncatus*) with paracoccidioidomycosis ceti, Indian River Lagoon, Florida, USA, showing typical branching chains of yeast-like cells of *Paracoccidioides brasiliensis* connected by small isthmuses. A) Strain FB-921; B) FB-938; C) FB-946; D) FB-952; E) B92-932; F) SW070458. Gomori’s methenamine silver stained. Scale bars indicate 10 µm.

Microscopically, the 6 silver-stained specimens showed branching chains of yeast-like cells connected by small isthmuses, which is typical of this pathogen from infected dolphins with lacaziosis/lobomycosis (Figure 1). PCR amplified the 151-bp DNA sequence from each of the genomic DNAs from the 6 dolphin formalin-fixed tissues. These DNA sequences were deposited into GenBank under accession nos. KX239500 for SW0704, KX239501 for FB946, KX239502 for FB921, KX239503 for FB 952, KX239504 for FB938, and KX239505 for B92-932. Primers targeting other DNA sequences >300 bp did not produce amplicons for all 6 DNA specimens.

**Figure 2:**
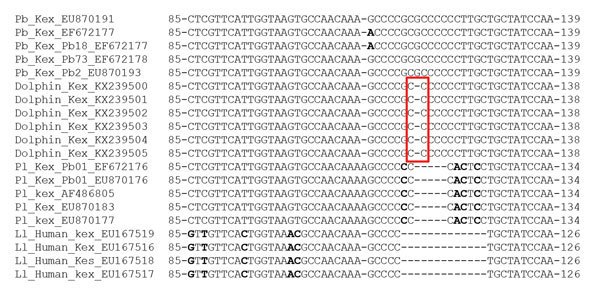
Nucleotide sequences of partial *Kex* gene exons of *Lacazia loboi* (Ll) and *Paracoccidioides brasiliensis* (Pb), including pathogen DNA sequences isolated from bottlenose dolphins, Indian River Lagoon, Florida, USA, and *P. lutzii* (Pl) containing mismatches (bold) and unique gaps. Red box indicates DNA sequences missing a nucleotide present in *P. brasiliensis* from humans. Numbers before and after sequences indicate nucleotide location of the depicted epitope. –, deletion.

Alignment of *P. brasiliensis* and *L. loboi* sequences from humans available in GenBank showed that partial *Kex* gene sequences of these fungi from dolphins were similar to those of *P. brasilienis* from humans. The only difference between *P. brasiliensis* sequences from humans and those from dolphins was a gap caused by a missing nucleotide in *P. brasiliensis* sequence from dolphins (Figure 2). *P. lutzii* and *L. loboi* sequences had several nucleotide mismatches and long gaps caused by several missing nucleotides (Figure 2). BLAST (https://blast.ncbi.nlm.nih.gov/Blast.cgi) analysis showed that the 6 partial *Kex* gene sequences had 100% homology with 7 *P. brasiliensis* sequences (GenBank accession nos. EU870193, EF672178, EF672177, EU870183, EU870177, EU870176, and EF672176), 93% homology with 5 *P. lutzii* sequences (GenBank accession nos. EF672176, EU870176, AF486805, EU870183, and EU870177), and 73% homology with 4 *L. loboi* sequences (GenBank accession nos. EU167516, EU167517, EU167518, and EU167519).

### Phylogenetic Analysis

**Figure 3:**
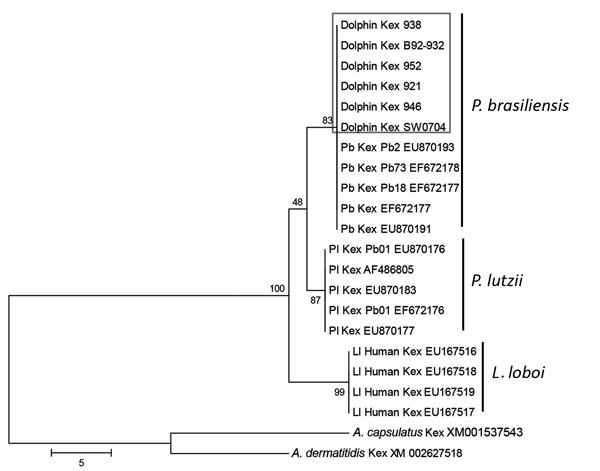
Unrooted maximum-parsimony phylogenetic tree of partial *Kex* gene sequences of *Paracoccidioides brasiliensis* (Pb) from 6 bottlenose dolphins, Indian River Lagoon, Florida, USA, with skin granulomas and homologous sequences of *P. brasiliensis*, *P. lutzii* (Pl), and *Lacazia loboi* (Ll) available in GenBank. *Ajellomyces capsulatus* and *A. dermatitidis* homologous sequences were used as outgroups. Strain names or accession numbers are shown. Numbers along branches are bootstrap values for 1,000 resamplings as obtained by parsimony analysis, which support different clusters. Box indicates uncultivated *P. brasiliensis* from dolphins grouped in the same cluster with cultivated *P. brasiliensis* from humans with paracoccidioidomycosis. Sequences of *P. lutzii* and *L. loboi* were placed with low bootstrap support as the sister group to *P. brasiliensis*. Scale bar indicates nucleotide substitutions per site.

**Figure 4:**
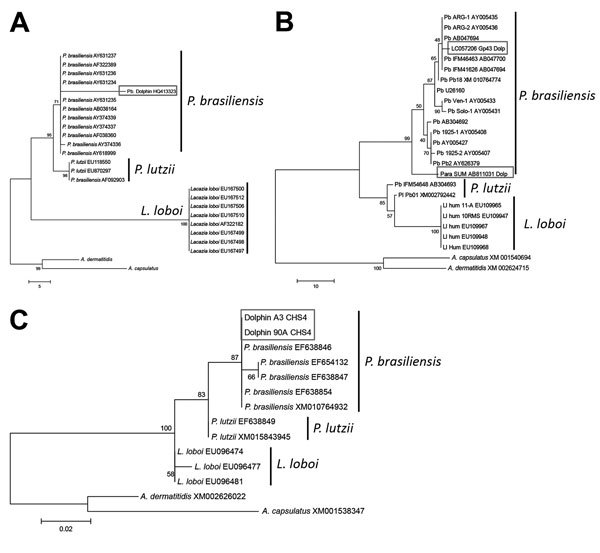
Unrooted maximum-parsimony phylogenetic trees of A) partial internal transcribed spacer (ITS), B) 2 partial glycoprotein 43 (*gp43*) (*12*–*14*), and C) 2 partial chitin synthase 4 (*CHS4*) (A. Schaefer, P.J. McCarthy, unpub. data) gene sequences of *Paracoccidioides brasiliensis*. Sequences were obtained pathogen-infected bottlenose dolphins, Indian River Lagoon, Florida, USA, and compared with homologous sequences of cultivated *Paracoccidioides brasiliensis* (*Pb*), *P. lutzii* (Pi), and uncultivated *Lacazia loboi* (Ll) available in GenBank. *Ajellomyeces capsulatus* and *A. dermatitidis CHS4*, *gp43*, and ITS homologous sequences were used as outgroups. Strain names or accession numbers are shown. Numbers along branches are bootstrap values for 1,000 resamplings obtained by parsimony analysis, which support different clusters. *P. brasiliensis* ITS sequences from dolphins (*14*) grouped among cultivated *P. brasiliensis* ITS sequences. Distance between uncultivated *P. brasiliensis* from dolphins and cultivated *P. brasiliensis* from humans is unusually large (box in panel A). Placement of 2 *gp43* *P. brasiliensis* sequences from dolphins (*12*,*13*) among cultivated *P. brasiliensis* strains (boxes in panel B). *P. brasiliensis* partial *CHS4* gene sequences from 2 dolphins placed these sequences (GenBank accession no. KX267767 [A3] and KX267768 [90A]) within the *P. brasiliensis* cluster (box in panel C). Scale bars indicate nucleotide substitutions per site.

Analysis of homologous partial *CHS4, Gp43*, *Kex*, and ITS sequences of *P. brasiliensis*, *P. lutzii*, *L. loboi*, *A. capsulatus*, and *A. dermatitidis* (the 2 *Ajellomyces* species sequences were used as outgroups) by parsimony and neighbor-joining showed that dolphin-derived pathogen sequences could be placed among *P. brasiliensis* sequences isolated from humans with paracoccidioidomycosis (Figure 3). *P. lutzii* and *L. loboi* resolved into 2 low-supported clusters. The partial *Kex* gene sequences of *L. loboi* available in GenBank placed this uncultivated pathogen among *Paracoccidioides* species (Figure 3). Placement of dolphin pathogen *Kex* gene sequences within the cluster of *P. brasiliensis* was also phylogenetically corroborated by using *CHS4*, *Gp43*, and ITS sequences available in GenBank (Figure 4). Dolphin-derived pathogen sequences clustered with good bootstrap support among sequences of *P. brasiliensis* isolates from humans.

## Discussion

We found that fungal DNA sequences isolated from dolphins with skin granulomas containing yeast-like cells had strong homology with sequences of cultivated *P. brasiliesis* from humans (*14*–*16*). Since cutaneous granulomas containing chains of yeast-like cells in 3 dolphin species (*Sotalia guainensis*, *T. aduncus*, and *T. truncatus*) were initially reported, the etiologic agent of lacaziosis/lobomycosis was believed to be *L. loboi*, which causes similar skin granulomas in humans (*1*–*7*,*19*). This hypothesis was based on phenotypic characteristics of the pathogen (uniform size yeast-like cells in chains connected by slender isthmuses and resistance to culture) and clinical presentation (keloidal-like granulomas) in humans and dolphins with lacaziosis/lobomycosis (*1*,*4*,*19*). Although some authors had reported minor phenotypic differences, such as smaller size of yeast-like cells in infected dolphins than of yeast-like cells in infected humans (*20*), the true phenotypic differences between the causative agent of keloidal-like skin infections in dolphins and humans are not fully understood (*4*,*20*).

Studies using serum samples from humans and dolphins with lacaziosis/lobomycosis, mice experimentally infected with *L. loboi*, and serum samples from humans with paracocidioidomycosis showed that IgG in serum samples from dolphins and humans infected with *L. loboi* had strong cross-reactivity with the gp43 antigen of *P. brasiliensis* (*4*,*6*,*21*). These findings support the hypothesis that the uncultivated organism causing cutaneous granulomas in humans and dolphins was *L. loboi*. Findings also implied that the gp43 antigen of the etiologic agent of parakeloidal-like granulomas in humans and dolphins was antigenically similar to that of *P. brasiliensis*. On the basis of these serologic studies (*4*,*6*,*21*), current phylogenetic data for *gp43* and *Kex* gene exons, and ITS DNA sequences, placement of *L. loboi* from humans in its own genus is questionable. Efforts to culture the organism from dolphins on classical laboratory media successfully used to isolate *P. brasiliensis* from humans with paracoccidioidomycosis were not successful (*4*,*7*). The physiologic basis of the inability to culture the etiologic agent from dolphins with cutaneous granulomas is not known. Thus, the life cycle features of this agent remain an enigma.

Our phylogenetic (parsimony) analysis of partial *Kex* DNA sequences validated reports suggesting that keloidal-like lesions in dolphins are caused by a novel uncultivated *P. brasiliensis* (*13*–*16*). We analyzed DNA sequences of pathogens isolated from 6 dolphins with lacaziosis/lobomycosis captured in the Indian River Lagoon. Diverse geographic locations of dolphins in the Atlantic Ocean (*13**,**16*) and the Pacific Ocean (*14*,*15*) and specimens evaluated by molecular methods provide additional support for placement of the etiologic agent of keloidal-like granulomas in dolphins within *P. brasiliensis* (Figure 3). Because these geographic locations, especially for cases from Japan (*14*,*15*), have different ecologic niches than locations for *P. brasiliensis* in South America (*4*), detection of dolphins infected with an uncultivated *P. brasiliensis* type in these ecosystems is a major finding.

Moreover, our phylogenetic data obtained by using *gp43* gene exons of Minakawa et al. (*14*) and Ueda et al. (*15*), ITS sequences of Esperón et al. (*16*), and 2 CHS4 gene sequences (A. Schaefer, P. McCarthy, unpub. data) strongly support placement of the dolphin pathogen within cultivated *P. brasiliensis* isolates from humans (Figure 4). The distance between ITS sequences from dolphins and *P. brasiliensis* ITS sequences from humans is large (Figure 4, panel A). An evaluation of additional ITS sequences from dolphin uncultivated *P. brasiliensis* strains from dolphins is needed to determine if this variation indicates 2 different populations or rapid substitutions in this DNA region.

Molecular data for dolphins in the Pacific and Atlantic Oceans in previous studies (*13*–*16*), the 6 pathogen DNA sequences isolated from dolphins (this study), and 2 *CHS4* gene sequences (A. Schaefer, P. McCarthy, unpub. data) place the uncultivated pathogen within cultivated *P. brasiliensis* strains. These studies added support to the notion that a novel uncultivated *P. brasiliensis*, which is different from the cultivated *P. brasiliensis* causing human paracoccidioidomycosis and *L. loboi* causing parakeloidal-like lesions in humans, is the causative agent of lacaziosis/lobomycosis in dolphins. Placement of *L. loboi* in a different cluster from dolphin-derived uncultivated *P. brasiliensis* indicates that, although both pathogens have identical phenotypes and cause similar skin lesions, they have different evolutionary paths.

Disease that shows keloidal-like granulomas in humans and dolphins has been known by several different names, such as Jorge Lobo disease (*4*), Lobo’s disease (*3*,*5*,*22*), lobomycosis (*1*,*6*,*13*,*16*,*19*,*23*,*24*), and lacaziosis (*11*,*12*,*14*,*15*). In view of most recent findings, the names used to describe this disease in dolphins are no longer supported. Minakawa et al. (*14*) proposed maintaining the name lacaziosis with the understanding that this name would include *L. loboi* (humans), uncultivated *Paracoccidioides* species, and *P. brasiliensis* (dolphins). However, in our phylogenetic analysis, the *Paracoccidioides* sp. strain (GenBank accession no. AB811031) of Ueda et al. (*15*) from an infected dolphin grouped among human *P. brasiliensis* strains. Thus, this strain is phylogenetically similar to strain LC057206. Furthermore, phylogenetic analysis of ITS sequences from dolphins with lacaziosis/lobomycosis placed *L. loboi* (with strong bootstrap support) in its own genus (Figure 3, panel A). Thus, the proposal by Minakawa et al. (*14*). could add more confusion to the taxonomic status of these 2 uncultivated fungal etiologies. In the interim, we propose paracoccidioidomycosis ceti for the disease caused by uncultivated *P. brasiliensis* in dolphins. This term best describes the current status of infected dolphins with keloidal-like granulomas and yeast-like cells in chains in infected tissues.

Uncultivated *P. brasiliensis* from Japan that causes skin infections in a new species of dolphins (*Lagenorhynchus obliquidens*) suggests that the geographic distribution of this pathogen is expanding and could also infect other species (*14*,*15*). Thus, whales and other cetaceans need to be investigated for this pathogen (*14*). Paniz-Mondolfi et al. (*24*) suggested that distinguishing apparent expansions of the ecologic niche caused by increased interest and surveillance by identification programs from a change in distribution would be difficult. The likelihood that this phenomenon is an expansion of its ecologic niche caused by global climate changes or increased surveillance is difficult to prove, but it is an intriguing possibility.

Although the ITS sequences of *L. loboi* from humans still group this pathogen in its own cluster, our molecular data for DNA protein-coding sequences indicate that the 3 species in this study (cultivated and uncultivated *P. brasiliensis*, *P. lutzii* from humans and dolphins, and *L. loboi* from humans) all have the same ancestor. Thus, all 3 species belong to the same genus (*Paracocidioides*). Comprehensive phylogenetic and genomic analyses of *L. loboi* from humans and uncultivated *P. brasiliensis* from dolphins are needed to corroborate results of these analyses and identify the true evolutionary history of *L. loboi* from humans. Our findings could stimulate new interest in lacaziosis and paracoccidioidomycosis ceti, which has been restricted to humans in South America and dolphins in many oceans.
